# Genome–epigenome crosstalk in T-cell lymphomas: from maps to mechanisms

**DOI:** 10.1007/s12185-025-04115-9

**Published:** 2025-11-26

**Authors:** Makoto Yamagishi

**Affiliations:** Laboratory of Viral Oncology and Genomics, Department of Computational Biology and Medical Sciences, Graduate School of Frontier Sciences, The University of Tokyo, Tokyo, Japan

**Keywords:** PTCL, Genetics, Epigenetics, EZH1/2

## Abstract

T-cell lymphomas are clinically and biologically heterogeneous malignancies that comprise ~ 10% of non-Hodgkin lymphomas. Outcomes with first-line chemotherapy remain poor. Over the past decade, integrative genomic and epigenomic studies have defined recurrent abnormalities converging on proximal T-cell antigen receptor/costimulatory signaling to the NF-κB/NFAT, JAK/STAT, PI3K/AKT/mTOR, and NOTCH pathways, alongside pervasive alterations in chromatin modifiers and the DNA methylation machinery. In this review, we frame the biology of peripheral T-cell lymphoma as two interdependent layers, including genetic events that establish constitutive signaling programs and epigenomic remodeling that stabilizes these outputs. We overview genomic alterations across major peripheral T-cell lymphoma entities and analyze epigenomic dysregulation, focusing on DNA methylation, enhancer regulation, and polycomb-mediated gene control. We highlight adult T-cell leukemia/lymphoma as a paradigmatic dual-layer disease, summarize therapeutic approaches based on epigenetic traits, and discuss biomarker-guided strategies and challenges in translating integrated maps into durable disease control.

## Introduction

T- and natural killer (NK)-cell lymphomas comprise a biologically and clinically heterogeneous group of mature T- and NK-cell neoplasms, which account for approximately 10% and 15%–20% of non-Hodgkin lymphomas in Western and East Asian cohorts, respectively [1–3]. Despite advances in supportive care and transplantation, first-line chemotherapy induces durable remission in only a minority of patients and relapse is typically aggressive, with few effective salvage options available [3, 4].

Over the past decade, integrative genomic studies have reshaped our understanding of the biology underlying T-cell lymphomas. Studies reveal that recurrent lesions converge on a limited set of signaling axes. T-cell antigen receptor (TCR) and costimulatory modules as proximal pathways signal to the nuclear factor kappa-light-chain-enhancer of activated B cells (NF-κB)/nuclear factor of activated T cells (NFAT), Janus kinase (JAK)/signal transducer and activator of transcription (STAT), phosphoinositide 3 kinase (PI3K)/AKT/mammalian target of rapamycin (mTOR), and the context-dependent NOTCH pathways, whereas alterations in chromatin modifiers, DNA methylation, and the enhancer architecture reprogram lineage identity and fitness.

Crucially, in the context of T-cell lymphoma, the genome and epigenome act as interdependent layers rather than competing concepts. Recurrent mutations, copy number alterations, and structural variants, including oncogenic fusions and noncoding disruptions, install constitutive signaling programs, whereas epigenomic remodeling, including DNA methylation imbalance, enhancer reprogramming, and polycomb repressive complex 2 (PRC2)-mediated H3K27 trimethylation, stabilizes these gene expression programs. This review provides a synopsis of genomic and epigenomic alterations in T-cell lymphomas and seeks to delineate future challenges based on the current landscape, with an emphasis on the development of therapeutic strategies highlighted by recent comprehensive advances.

## Genomic alterations in peripheral T-cell lymphomas

**Fig. 1:**
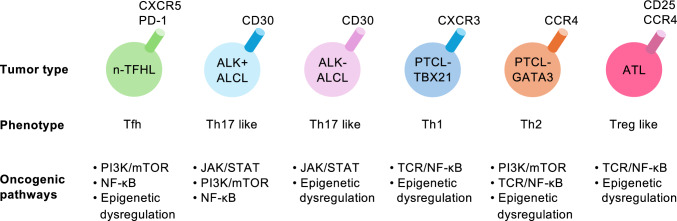
Major PTCL subtypes and association with states/cell-of-origin and genetic alteration. Summary schematic of key cell surface markers, cellular phenotypes, and major oncogenic pathways across PTCL subtypes. These phenotypic features and genetic alterations are not mutually exclusive; intermediate states, overlap, and phenotypic plasticity are observed

Peripheral T-cell lymphomas (PTCLs) comprise a biologically diverse group of mature T-cell neoplasms, in which recurrent genomic abnormalities converge on the proximal TCR/CD28 signaling, with downstream effectors (RAS/ERK and PI3K/AKT), RHO family of GTPases, and the activation of JAK/STAT signaling (Fig. 1). Contemporary PTCL classification by the World Health Organization and the new International Consensus Classification emphasizes the cell of origin and the state of differentiation, although integrated genomic studies increasingly refine diagnostic boundaries and expose targetable vulnerabilities [3, 5]. PTCLs share a recurring architecture involving epigenetic derangements and mutations impinging on the proximal TCR/CD28 signaling and the downstream JAK/STAT pathway, with the tripartite pattern currently framing both the biology and therapeutic considerations.

## Nodal follicular helper T-cell lymphoma (n-THFL)

Nodal follicular helper T (Tfh) cell lymphoma (n-TFHL), which encompasses angioimmunoblastic T-cell lymphoma (AITL) and related subtypes, is characterized by lesions that either prime the Tfh cell program or amplify the antigen receptor costimulation. Canonical examples include the R172 mutation of *IDH2*, which defines a molecular subset, and frequent mutations in *TET2* and *DNMT3A*, which sometimes present as clonal hematopoiesis in nontumor hematopoietic compartments [6–11]. IDH2 and TET2 cooperate to shape Tfh cell function and the tumor microenvironment in experimental systems, providing a biological explanation for their recurrent cooccurrence [12]. In recurrent n-TFHL, *RHOA* G17V functions as a lineage-directing hit, promoting Tfh cell specification and lymphomagenesis in mouse models and patient cohorts [11, 13–17].

Upstream of TCR signaling, tumors deploy multiple means to boost costimulatory signals. Activating mutations in *CD28* and fusions involving *CD28*, such as *CD28::CTLA4* and *CD28::ICOS*, enhance CD28-mediated signaling. Additional TCR-proximal fusions activate nonreceptor tyrosine kinases, such as those observed with lesions harboring *FYN::TRAF3IP2* or *KHDRBS1::LCK*, or induce guanine nucleotide exchange factor signaling, such as that observed with lesions exhibiting VAV1 activation, all mimicking chronic T-cell activation [18–20]. Likewise, the *ITK::SYK* fusion, a paradigmatic lesion originally described in Tfh-type disease and functionally modeled in mice, delivers constitutive TCR-like signals [18, 19]. Together, these alterations rationalize a disease model in which mutations in Tfh cell programming and alterations in tonic antigen receptor signaling are complementary oncogenic modules [2, 12, 20, 21].

## Anaplastic large cell lymphoma (ALCL)

ALK-positive anaplastic large cell lymphoma** (**ALCL) is driven by *ALK* fusions, most commonly *NPM::ALK* arising from t(2;5)(p23;q35). These fusions constitutively induce ALK activity and the JAK/STAT3, RAS/ERK, and PI3K/AKT cascades, among others. The efficacy of pharmacologic ALK inhibition has been shown in relapsed/refractory ALCL, providing strong genetic–therapeutic concordance [22, 23]. Transcriptionally, ALK-positive ALCL exhibits a STAT3-regulated profile and distinct mRNA signatures, wherein the cooperation between the AP-1/BATF family and IRF4 sustains tumor cell proliferation and the ALCL identity [24, 25].

Despite the homogenous morphology and comparable CD30 expression levels, ALK-negative ALCLs are genetically heterogeneous. Two recurrent rearrangements stratify patient outcomes, with *DUSP22* and *TP63* rearrangements considered favorable and unfavorable features, respectively. Typically, these two lesions are mutually exclusive. Moreover, some patients harbor *STAT3* or *JAK1* mutations and fusions involving transcription factors, such as *NFKB2* and *NCOR2*, and kinases, such as *ROS1* and*TYK2*, which converge on STAT3 activation as a common oncogenic node [26–29].

## PTCL, not otherwise specified (PTCL-NOS)

PTCL, not otherwise specified (PTCL-NOS) is a heterogeneous group of tumors that lack the defining entity-level lesions but can be molecularly stratified. Cytogenetic studies often reveal complex karyotypes with recurrent gains of 7q/8q, and proposed targets in chromosome 7 include *CDK6* and *CARD11* [30, 31]. *TP63* rearrangements are detected in < 10% of the patients with PTCL-NOS [32]. Genomically unstable PTCL-NOS with *TP53* or *CDKN2A* alterations confer poor prognosis [33], whereas whole-genome sequencing highlights the codeletion of *CDKN2A* and *PTEN* as a characteristic feature of PTCL-NOS [34]. Commonly altered genes include epigenetic regulators, *FAT1*, *TP53*, and genes involved in immune surveillance [2, 35]. Signaling pathways are frequently rewired by VAV1 activation through multiple fusions or splice variants and by rarer fusions, such as *FYN::TRAF3IP2*, *KHDRBS1::LCK*, and those involving *CD28* [2, 35].

Gene expression profiling of PTCL-NOS has identified two principal molecular signatures, namely the TBX21 subgroup with T helper type 1 cell-like signature, which is enriched for NF-κB signaling, and the GATA3 subgroup with a T helper type 2 cell-like signature, which is characterized by MYC/proliferation signatures and adverse outcomes [36, 37]. Within the TBX21 subgroup, a cytotoxic subset with *DNMT3A* mutations exhibits distinct biology and prognosis [38]. TBX21 tumors exhibit lower genomic complexity with more frequent epigenetic dysregulation involving *TET2* and *DNMT3A*, whereas GATA3 tumors accumulate deletions in *TP53*, *CDKN2A*, *PRDM1*, and *PTEN*, consistent with impaired genomic surveillance and restraint of the PI3K pathway [1, 2].

## Adult T-cell leukemia/lymphoma (ATL)

Adult T-cell leukemia/lymphoma (ATL) is a mature T-cell malignancy that arises in the context of HTLV-1 infection and is clinically categorized into acute, lymphoma, chronic, and smoldering forms [39]. Integrated genomic analyses of ATL reveal the constitutive activation of TCR and costimulatory receptors and the NF-κB pathway as disease drivers, coupled with genetic hits that amplify tissue homing and immune evasion [40]. Representative alterations are also observed in genes involved in immune evasion (*PD-L1*, *HLA class I*, *CD58*, and *FA*S) and T-cell trafficking (*CCR4*, *CCR7*, and *GPR183*), transcription factors and repressors that are essential for lymphocyte function (*IKZF2*, *GATA3*, *IRF4*, and *CIC*), epigenetic regulators (*TET2*, *DNMT3A*, and *EP300*), and DNA repair genes (*TP53*, *CDKN2A*, and *POT1*).

Recurrent alterations affect TCR-proximal signaling and downstream scaffolds, including *PLCG1*, *PRKCB, CARD11, VAV1*, and *IRF4*, and collectively enforce tonic receptor signaling with NF-κB activation [40]. These events provide a genetic scaffold that explains the persistent activation phenotype and cytokine milieu that are typically observed in ATL. CD28 signaling is further augmented by copy number amplifications and activating point mutations in younger patients with ATL. CD28 fusions, such as *CD28::CTLA4* and *CD28::ICOS*, are recurrent and expand the costimulatory output [40, 41]. Such fusions, which neatly align with the broader theme of TCR-proximal rewiring through mutations and structural variants observed in patients with PTCL, are conversely superimposed on a viral background that favors survival and proliferation in patients with ATL.

Gain-of-function mutations in *CCR4*, and to a lesser extent in *CCR7*, truncate the C-terminal cytoplasmic tail of the protein, impair ligand-induced internalization, and enhance chemotaxis, thereby reinforcing leukemic trafficking and microenvironmental engagement [40, 42]. Clinically, mogamulizumab, an anti-CCR4 monoclonal antibody, exhibits efficacy in relapsed ATL, with responses particularly notable in patients with *CCR4* mutations, mechanistically consistent with receptor hyperactivity [43]. Disruptions in the 3’ untranslated region of *PD-L1* (*CD274*) stabilize transcripts and induce cell surface expression, offering a direct structural route to immune escape [44]. This mechanism has been described across cancers and is specifically relevant in T- and NK-cell neoplasms, including virus-associated settings.

Cohort comparisons underscore the geographic diversity of the mutation spectrum and therapeutic sensitivity. A study including a North American cohort reported a distinct mutational/transcriptional profile with responsiveness to epigenetic therapies, compared to the patterns observed in some Japanese cohorts, highlighting the importance of the population context in trial design and biomarker deployment [45]. In addition, studies reveal that clonal evolution precedes clinical onset and that the stepwise accrual of somatic mutations is already detectable even in HTLV-1 carriers without ATL [46, 47]. These observations indicate that recurrent genetic lesions are not merely associated with the ATL phenotype and pathobiology but represent positively selected driver events that confer clonal fitness and promote outgrowth.

## Epigenomic dysregulation in PTCL: one system built from three layers

Although PTCL harbors entity-specific genetic hallmarks, epigenomic dysregulation is pervasive and encodes a malignant memory. In a manner consistent with efforts to stratify PTCL based on genetic lesions, DNA methylation, histone acetylation (i.e., enhancer architecture), and polycomb-mediated H3K27 methylation should be considered the three interlocking epigenomic features of PTCL.

DNA methylation is profoundly perturbed in Tfh-type PTCL, as reflected in the high prevalence of *TET2, DNMT3A*, and *IDH2* mutations in these patients. Across the genome, promoters of immune modulators and lineage-governing genes accrue aberrant hypermethylation signatures, whereas broader hypomethylation can destabilize chromatin organization [1, 2, 47, 48]. In ATL, the extensive hypermethylation of CpG islands substantially contributes to the distinctive transcriptional reprogramming of tumor cells [40, 49]. These features have therapeutic implications. Hypomethylating therapy can reactivate programs silenced by DNA methylation and has been demonstrated to provide encouraging results when utilized in combination with chemotherapy in patients with Tfh-type PTCL [50–52].

Enhancer acetylation, which is typically indexed by H3K27ac marks, underpins lineage identity and proliferative circuitry. Across PTCL subtypes, super-enhancers nucleate around master transcription factors and survival drivers, and their redistribution aids in establishing disease-specific expression patterns and furnishes mechanisms for cellular plasticity [53, 54]. Histone deacetylase (HDAC) inhibitors modulate this acetylation-centered enhancer activity, inducing differentiation and apoptosis. Clinically, responses tend to concentrate within Tfh phenotypes, in agreement with their epigenetic redisposition [55, 56].

Gene repression via polycomb repressive complex 2, reflected by the H3K27me3 mark, is a powerful silencing mechanism across PTCL types and has been extensively delineated in ATL. Genome-wide profiling demonstrates the de novo accumulation of H3K27me3 across multiple loci encoding tumor suppressors, immune response genes, and differentiation regulators [57, 58]. A key consideration in lymphoid cells is the coexpression of enhancer of zeste homolog (EZH) 1 and 2. Whereas EZH2-selective inhibition often leads to residual methylation sustained by EZH1, dual EZH1/2 blockade is more effective in the complete depletion of H3K27me3 and efficiently reawakens gene sets that are selectively silenced in tumor cells [59, 60]. Beyond these direct chromatin effects, the loss of H3K27me3 is also predicted to retune enhancer–promoter communication.

These three genome-wide epigenetic features are not independent. Chronic activation of signaling pathways, often initiated by genetic hits, reshapes transcription factor occupancy and enhancer selection, thereby feeding back on signaling output. Although the enzymes and protein complexes that are involved differ, they interfere with and, in certain contexts, directly interact with each other to determine the cellular epigenomic state [61]. Taken together, the histone code and DNA methylation constitute an “epigenomic code” that orchestrates the spatiotemporal control of genomic loci. Since the phenotypic expression of genomic lesions is ultimately filtered through epigenomic regulation, a faithful account of tumor identity, clonal evolutionary mechanisms, rational therapy design, and precise risk assessment requires the consideration of multi-dimensional genome regulation as a coupled complex system.

## ATL as a paradigmatic genetic/epigenetic dual-layer disease

**Fig. 2:**
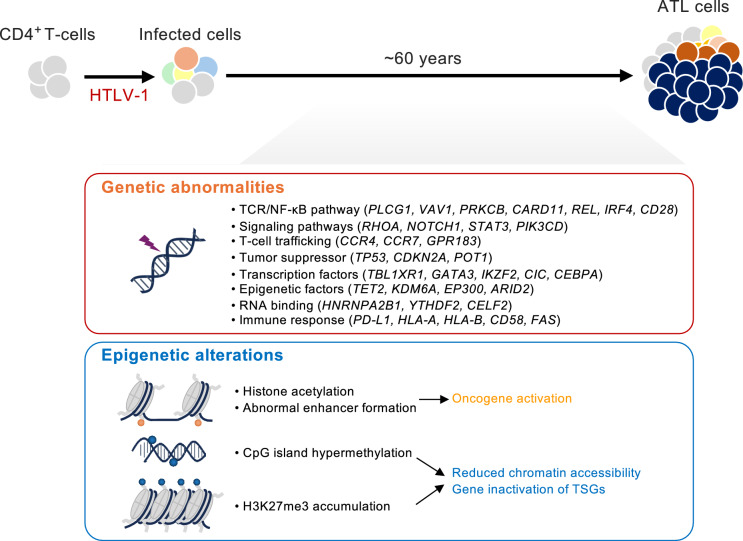
Genome–epigenome crosstalk in ATL. During the prolonged latency following HTLV-1 infection, malignant ATL clones emerge as several of the depicted abnormalities undergo clonal selection via as-yet-unknown mechanisms. Genomic and epigenomic alterations are interdependent; neither layer alone fully accounts for ATL phenotypes or pathogenesis

ATL is an aggressive T-cell malignancy that arises following the retroviral disruption of host genes, with the convergence of a protracted multistep oncogenic process and entrenched genomic and epigenomic aberrations, offering a window into carcinogenesis (Fig. 2). In important respects, the polyclonal cell population created by the random insertion of the HTLV-1 provirus into the host genome resembles the mosaic clonal architecture laden with innumerable somatic lesions observed in precancerous solid tissues. Disease emergence is driven by the expansion of selective clones. Cross-sectional and longitudinal sequencing studies indicate that driver mutations and copy number alterations accumulate over time, tracing patient-specific evolutionary trajectories [47]. Yet, the aggregate evaluation of multiple specimens reveals recurrent pathway-level lesions. The apparently stochastic catalog of mutations obeys discernible rules, and one can, from vast datasets, infer the selective ascent of ancestral clones that likely emerged decades earlier [40, 47, 62].

In contrast, the epigenome of ATL displays striking consistency, although case-to-case characteristics are evident at the level of individual genes. Genome-wide studies reveal broad gains of H3K27me3, which silence numerous genes, prominently tumor suppressors, in combination with a tumor-specific regulatory network built on this foundation. Functionally, ATL cells depend on polycomb-mediated control sustained by both EZH2 and EZH1. In addition, these cells exhibit enhancer rewiring that supports highly proliferative compartments and promoter-centered DNA methylation that consolidates repression. Recent studies also suggest that HTLV-1 infection leads to early perturbations in host chromatin architecture and the enhancer landscape and that an epigenetic memory of this insult can persist even after the decline of viral gene expression [63, 64].

Insights from genomic and epigenomic studies are being translated into therapeutic efforts. From a genomic standpoint, biomarkers such as *CCR4* mutation/expression and *PD-L1* 3’UTR disruption are under discussion as predictors of benefit for antibody-based and immune-directed strategies in defined contexts [43, 44]. From an epigenomic standpoint, the clinical utility of the dual EZH1/2 inhibitor valemetostat as a single-agent therapy has been demonstrated in relapsed/refractory ATL, with pharmacodynamic evidence of H3K27me3 depletion and gene reactivation, consistent with the underlying mechanism [60]. Furthermore, the widespread reprogramming of gene expression, established by genetic/epigenetic variations, is tightly linked to its phenotypic and functional hallmarks regarding the therapeutics. Activation of the GARP/TGF-β axis enforces commitment to the Treg cell lineage, suppressing the activity of neighboring effector T cells while concurrently promoting the proliferation of ATL cells. Targeting the GARP/TGF-β axis may aid in resolving both the proliferative drive of tumor cells and the immune evasion [65]. Going forward, efforts in ATL research should integrate these multiple layers to accelerate the biologic understanding of disease and the development of therapeutics.

## Therapeutic landscape and clinical integration in PTCL

Currently employed treatment approaches in PTCL extend beyond conventional chemotherapy and encompass epigenetic agents and pathway-directed therapies. The most clinically consequential epigenetic therapeutic modalities are HDAC inhibitors, DNA methylation-targeted therapy, and EZH1/2 blockers.

HDAC inhibitors induce broad transcriptional reprogramming, culminating in differentiation, apoptosis, and enhanced immune visibility. Prospective and retrospective studies reveal that responses to HDAC inhibitors tend to be enriched in Tfh phenotypes. HDAC inhibitors can be deployed as front-line treatment in combination with the CHOP regimen or as salvage therapy [66, 67].

DNA methylation-targeted therapies aim at the methylation axis central to TFHL. Oral azacitidine plus CHOP has yielded high complete response rates overall, with particularly notable efficacy in Tfh-type PTCL. Likewise, combining hypomethylating therapy with HDAC inhibition is promising, reflecting convergence on enhancer–promoter communication and reexpression of immune-related genes [51, 68]. This coherence links the genomic context (e.g., mutations in *TET2*, *DNMT3A*, and *IDH2*) with the epigenetic mechanisms.

The dual EZH1/2 inhibitor valemetostat, whose utility has been mechanistically established in ATL, is being deployed as a therapeutic option in patients with PTCL. By achieving a more complete removal of H3K27me3 marks compared with EZH2-selective agents, valemetostat restores silenced networks and retunes transcription toward physiological states, as clinically evidenced by its effective single-agent activity in relapsed/refractory ATL and, more recently, in relapsed/refractory PTCL irrespective of the histologic type [69, 70]. The ensuing reverse translational work supports the view that polycomb dependency extends beyond ATL to PTCL-NOS, Tfh-type PTCL, and ALCL.

Given these complexities, the therapeutic framework should take the next step. To sustain benefit in tumors prone to resistance, bivariate biomarker strategies are advisable whenever feasible, by anchoring treatment on pathway lesions (e.g., JAK/STAT pathway and TCR-proximal activating mutations) and the epigenomic state (e.g., baseline H3K27me3 level and enhancer acetylation patterns). Adaptive sequencing approaches can stratify patients in real time, based on molecular readouts, with mutation panels serving as an entry point to precision therapeutics.

Epigenetic combinations are similarly compelling. The observation that resistance to EZH1/2 inhibitors may be accompanied by de novo DNA methylation under therapeutic pressure underscores multilayered maintenance as a core property of the tumor epigenome. As elegantly demonstrated by combining multiple antiretroviral drugs to achieve a functional cure in human immunodeficiency virus 1 (HIV-1), coordinated multi-drug strategies can be transformative in PTCL. With the detailed understanding of the complex genomic and epigenomic landscape of PTCL, it is time to contemplate the next move.

## Future directions

A central lesson of epigenetic therapy is the complexity of the mechanisms of action and resistance. For histone methylation-targeted approaches, emerging themes include EZH2/EZH1 redundancy, bypass via H3K27me3 and DNA methylation, and highly plastic states that modulate sensitivity [60]. Translational priorities should be logically derived from the already elucidated genomic and epigenomic maps.

Technological advances, especially single-cell methodologies, are revealing new features of PTCL. Transcriptional programs unveil significant therapeutic opportunities, whereas intra-tumoral diversity across the genome and epigenome poses a major challenge in durability. In particular, the detection of minimal residual disease using cell-free DNA and similar assays is poised to impact care, with further breakthroughs expected in the near future [71, 72]. The overarching goal is durable disease control by narrowing evolutionary exits through the neutralization of dominant pathway outputs while rewiring the epigenome to shrink the landscape of viable malignant states.

In summary, the biology of PTCL converges on the genome and epigenome as two equivalent, interlocked layers. Neither layer alone suffices to explain disease behavior, and clinical opportunities lie in organizing diagnosis, prognosis, and therapy around the interaction between the epigenome and genome.

## Data Availability

This article does not include any originial experimenta data.
